# Comprehending B-Cell Epitope Prediction to Develop Vaccines and Immunodiagnostics

**DOI:** 10.3389/fimmu.2022.908459

**Published:** 2022-07-07

**Authors:** Salvador Eugenio C. Caoili

**Affiliations:** Biomedical Innovations Research for Translational Health Science (BIRTHS) Laboratory, Department of Biochemistry and Molecular Biology, College of Medicine, University of the Philippines Manila, Manila, Philippines

**Keywords:** B-cell epitope prediction, B-cell epitopes, surface accessibility, conformational disorder, immunodominance, peptide-based vaccines, antipeptide antibodies, protective immunity

## 1 Introduction

B-cell epitope prediction (BCEP) is the original subject of immunoinformatics (i.e., bioinformatics applied to immunology). This began with protein sequence analysis to identify hydrophilic peptide fragments bound by antibodies that recognize whole proteins (1), to enable the earlier proposed development of synthetic peptide-based vaccines for inducing protective antibody-mediated immunity (2). BCEP was thus initially “prediction of protein antigenic determinants,” with each antigenic determinant being a B-cell epitope (BCE): a structural feature (e.g., sequence segment) recognized by a paratope (i.e., antigen-binding site of an immunoglobulin such as an antibody) (3, 4). Now understood as the computational identification of putative BCEs, BCEP has since grown to comprise much more sophisticated methods for analyzing both sequence (5–9) and higher-order structure (10, 11) on ever larger scales (e.g., applying genomics and proteomics for vaccine design (12, 13)). However, the full potential of BCEP for peptide-based vaccine design remains to be realized, for which reason the utility of BCEP as such has been called into question (14–17). Nevertheless, BCEP can support the development of vaccines and immunodiagnostics provided that its limitations are adequately comprehended and addressed.

## 2 Accessible Disorder (Ad) and Immunodominance

Accessible disorder (AD) is the state of a BCE that is simultaneously both paratope-accessible and disordered (i.e., conformationally unconstrained), such that BCE-paratope binding can occur *via* induced fit and/or conformational selection (8). Clearly, BCEs must be accessible to paratopes for physical contact to occur between them. In the context of antibody-mediated protective (e.g., antipathogen) immunity, this is most readily feasible for BCEs that are on outwardly protruding solvent-accessible molecular surfaces at extracellular sites (e.g., among secreted biomolecules). Hence, vaccine-design initiatives tend to selectively apply BCEP with a focus on surface-exposed sites among biomolecular targets of the pertinent (e.g., pathogen) secretome (i.e., totality of secreted biomolecules) and surfome (i.e., surface proteome) (18, 19). Additionally, surface complementarity must be attained between BCEs and their corresponding paratopes upon physical contact if stable BCE-paratope binding is to occur, for which the BCEs must adopt suitable conformations. This is favored where the BCEs are disordered prior to binding by the paratopes (20, 21). Vaccines can thus be produced from peptides comprising disordered BCEs of selected target proteins (e.g., pathogen virulence factors), to elicit production of antipeptide antibodies (i.e., peptide antibodies (22)) that can neutralize the biological activity of the proteins (4, 23–36). Such an approach is viable where the BCEs are disordered in both the peptides and the proteins; but if the BCEs are conformationally constrained (e.g., folded) in the proteins, their binding by the antipeptide antibodies may fail to occur, as is thought to be the case among unsuccessful attempts at peptide-based vaccine development (16).

**Figure 1:**
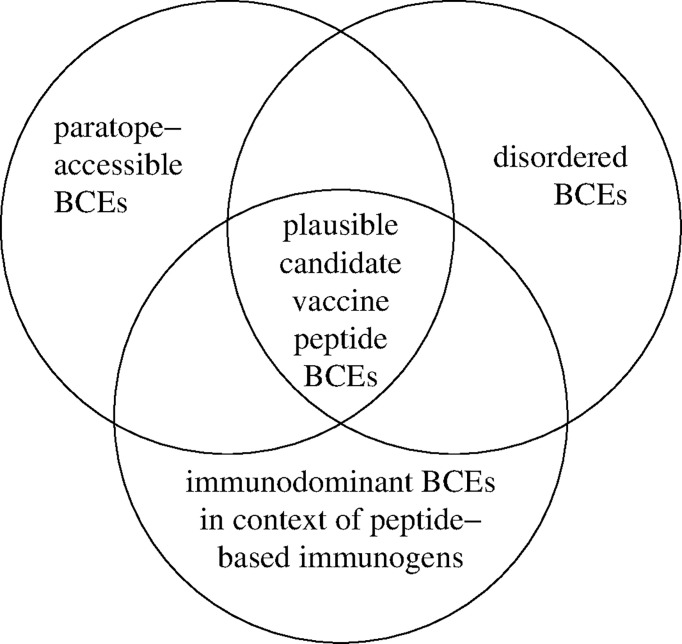
Identification of plausible candidate vaccine peptide BCEs. Accessible disorder (AD) is realized for BCEs that are simultaneously both paratope-accessible and disordered (i.e., conformationally unconstrained) in both peptide-based immunogens and cognate native antigenic targets *in situ* (e.g., extracellular pathogen virulence factors). Immunodominant BCEs are identified empirically as they occur in peptide-based immunogens (e.g., using immunogenic carrier molecules and immunologic adjuvants) versus other contexts (e.g., in native antigenic targets comprising antigenic decoys).

AD among BCEs thus facilitates BCE-paratope binding; but BCE-specific antibody production is also subject to the phenomenon of immunodominance (i.e., bias of immune responses toward subsets of BCEs encountered in the course of immunization), as depicted in **Figure 1**. Driven by Darwinian competition among B-cell clones, immunodominance tends to be favored by greater numbers of functional BCE-recognizing precursor B cells as well as stronger binding of BCEs by B cells in terms of both affinity (i.e., strength of binding per individual BCE-paratope pairwise interaction) and avidity (i.e., overall strength of cooperative binding among paratopes that simultaneously bind two or more BCEs on a single antigen, as is possible with engagement of one or more bivalent immunoglobulin molecules) (37). Consequently, individual host life history of antigenic exposure (e.g., *via* infection and immunization) influences immunodominance. Immunodominance may thus be precluded by immune tolerance (i.e., selective inability to mount immune responses to particular BCEs, due to functional deletion or inactivation of their corresponding B cells), which is often induced by BCEs of host self antigens (i.e., autoantigens) and of other antigens (e.g., in food) to which the host has been exposed in a natural physiologic setting (rather than in the course of infectious disease or vaccination) (38–40). Alternatively, immunodominance may be heightened by the immunological memory of prior immunization (e.g., *via* infection or vaccination), as occurs in the phenonenon of original antigenic sin (i.e., antigenic imprinting) whereby memory B-cell clones generated by past immunization continue to dominate antibody responses to more recent immunizations, possibly even compromising the ability to mount protective immune responses against newly encountered pathogen variants (41, 42). From an evolutionary standpoint, pathogens may co-evolve with their hosts to evade immune destruction in part by altering their BCE repertoires to limit the expression of immunodominant pathogen BCEs on key virulence factors (e.g., *via* molecular mimicry, with pathogen BCEs tending to resemble host self BCEs) while possibly also expressing immunodominant pathogen BCEs that serve as antigenic decoys to detract from protective host immune responses (43). Furthermore, immune tolerance may be broken under certain circumstances (e.g., infection by a pathogen employing molecular mimicry), which may result in antibody-mediated (e.g., autoimmune) disease (44). These various scenarios highlight the potential complexity of vaccine development with the diversity of BCEs and possible immune responses thereto. Peptide-based vaccine design thus provides opportunities to systematically restrict the repertoire of vaccine BCEs and thereby selectively target key biomolecules (e.g., critical virulence factors) while avoiding harmful or otherwise counterproductive antibody responses (e.g., to BCEs of autoantigens and antigenic decoys).

## 3 Toward New Vaccines and Immunodiagnostics

In essence, BCEP consists of two steps: structural partitioning of a prospective target (e.g., protein) into plausible candidate BCEs (e.g., peptidic sequences) and evaluation of these to assign them numerical scores that can inform subsequent decisions (e.g., on selecting components for inclusion in vaccines). Ideally, the scores would directly quantify functional impact (e.g., degree of antibody-mediated host protection against a protein toxin). In practice, functional impact can be estimated from BCE-paratope binding affinity in conjunction with a limited set of other key parameters (e.g., concentrations of antibody and its target), with said affinity itself being estimated as the BCE-paratope standard free-energy change of binding (∆*G*_b_°) based on target structure (8). This can be simplified by considering only candidate BCEs of paratope-accessible disordered target regions. Viewed more comprehensively, BCEP can be performed largely by excluding target regions that are at least partially inaccessible to paratopes. Such regions may be inaccessible due to biomolecular folding, complex formation (e.g., oligomerization) and anatomic compartmentalization (e.g., due to biomembranes). Accordingly, target regions may be identified for exclusion using appropriate computational tools to predict folding (vis-à-vis disorder, e.g., using AlphaFold (45, 46)) and higher-order structural organization (e.g., among supramolecular assemblies such as biomembranes (47)). For convenience, inaccessibility may be generalized to also include forms of posttranslational modification resulting from covalent linkage of nonprotein moieties (e.g., glycosylation), which can be computationally predicted to mark candidate BCEs for exclusion as well. Likewise, generalization of inaccessibility can also be extended to regions featuring disulfide-bond formation between cysteine residues, as this may impede BCE-paratope binding. After exclusion of implausible or otherwise potentially problematic target regions based on anticipated inaccessibility, ∆*G*_b_° and in turn functional impact may be estimated for the remaining candidate BCEs.

For the most part, BCEP can thus be regarded as prediction of AD. This varies with the envisioned practical application (e.g., vaccines versus immunodiagnostics). For vaccines, AD is defined by what can be achieved *in vivo* based on endogenous antibody production, with intracellular targets tending to be inaccessible under physiologic conditions, though antibodies are sometimes internalized by host cells in either free or pathogen-bound form to mediate immunity within certain intracellular compartments (48, 49). Such limitation may be overcome *via* immunotherapeutics using exogenously supplied antibodies and derivatives thereof (e.g., antibody fragments), notably with artificially produced cell-penetrating antibodies that can cross plasma membranes to bind intracellular targets (50, 51). For immunodiagnostics, the potential extent of AD is even greater, as constraints *in vivo* (e.g., on temperature and chemical composition) can be transcended *in vitro* (e.g., within a diagnostic test kit). For instance, membrane permeabilization (e.g., using detergents and/or organic solvents (52)) can extend paratope access into virtually all intracellular compartments; and treatment with chaotropic agents (e.g., urea (53) or ammonium thiocyanate (54)) can disrupt both intermolecular and intramolecular interactions, thereby increasing AD *via* order-to-disorder transitions, as in biomolecular disassembly and unfolding. BCEP itself is expanding in scope to support other applications, notably the design of novel antibodies and related constructs. BCEs can thus be identified initially as targets for binding by complementary peptides, which may be computationally designed and subsequently grafted on an antibody scaffold to produce novel antibodies that recognize the BCEs (55). The complementary peptide sequences might themselves be disordered, possibly remaining so on the antibody scaffold and even after binding their targets; but such persistent disorder could pose challenges for biotechnological antibody production, particularly with disordered regions that are recognized to initiate proteasome-mediated degradation (56). Nevertheless, said degradation could be circumvented *via* cell-free antibody production, which can also be used to produce cell-penetrating antibodies (57). Additionally, preexisting (e.g., antihapten) antibodies could be used with a diverse repertoire of peptide-based adaptors (e.g., hapten-labeled complementary peptides) for chemically programmable immunity that entails redirection of the antibodies to particular targets according to the specific choice of adaptors (58). Such repurposing of preexisting antibodies could enable more rapid responses to emerging threats (e.g., novel pathogens) than would be possible *via* development of novel antibodies or similar constructs.

Beyond predicting AD, BCEP also encompasses the more computationally challenging task of predicting conformational BCEs (i.e., BCEs that are to at least some extent conformationally constrained). Among folded proteins, conformational BCEs are surface-exposed regions that each constitute a paratope footprint and are thus typically discontinuous BCEs in the sense of comprising paratope-contacting residues that are noncontiguous along the protein sequence, sometimes even on separate polypeptide chains where proteins form supramolecular complexes such as viral capsids (3). Whereas prediction of AD can be cast as sequence profiling that is reminiscent of the earliest BCEP methods, BCEP for conformational BCEs entails the nontrivial cascading problems of delineating discontinuous candidate BCEs, predicting their hierarchy of immunodominance and estimating ∆*G*_b_° for cross-reactive binding of their corresponding paratopes to disordered candidate BCEs (e.g., peptidic sequences forming parts of discontinuous candidate BCEs) (59). This is further complicated by possible protein unfolding *in vivo* and consequent uncertainty regarding relevant candidate BCEs, which confounds interpretation of data on binding of antiprotein antibodies to peptide fragments of the cognate protein antigens (60). In light of these considerations, the apparently poor performance of BCEP methods benchmarked against said data (15, 61) is unsurprising and likely reflects the unmitigated complexity of factors underlying BCEP to identify short peptide sequences that are recognized by antiprotein antibodies. Yet, such challenges pose barriers to development of peptide-based constructs as immunodiagnostic reagents for detecting antiprotein antibodies rather than as vaccine components for eliciting production of antipeptide antibodies (59), though this crucial distinction was unfortunately obscured at the inception of BCEP (1). Confusion has thus resulted mainly from failure to distinguish between capacity of peptides to be bound by antiprotein antibodies (i.e., cross-reactive antigenicity) and capacity of peptides to elicit production of antipeptide antibodies that cross-react with proteins to confer protective immunity (i.e., cross-protective immunogenicity); and this has been the main reason underlying failed attempts at peptide-based vaccine development (17).

## 4 Conclusion

BCEP can be framed mainly as computational identification of putative paratope-accessible disordered peptidic sequences in an appropriate translational context (e.g., *in vivo* versus *in vitro*), thereby transcending limitations of earlier approaches to BCEP. This enables development of vaccines and immunodiagnostics, most notably by selecting BCEs for inclusion among peptide-based constructs that elicit production of antipeptide antibodies. Such antibodies can mediate protective immunity (e.g., by neutralizing pathogen virulence factors *in vivo*) and/or be used for antigen detection (e.g., with the aid of surfactants and other chaotropic agents to increase the extent of AD among pathogen-derived proteins *in vitro*). BCEP thus supports the design of peptide-based constructs that are potentially useful as vaccine components, as companion immunodiagnostics for monitoring antipeptide antibody responses to vaccination, and as means for generating antipeptide antibodies that, apart from mediating protective immunity (e.g., *via* active or passive immunization), may serve as immunodiagnostic reagents for antigen detection.

## Author Contributions

The author confirms being the sole contributor of this work and has approved it for publication.

## Funding

This work was funded by the University of the Philippines System, via One UP professorial chair grant 2019-100965.

## Conflict of Interest

The author declares that the research was conducted in the absence of any commercial or financial relationships that could be construed as a potential conflict of interest.

## Publisher’s Note

All claims expressed in this article are solely those of the authors and do not necessarily represent those of their affiliated organizations, or those of the publisher, the editors and the reviewers. Any product that may be evaluated in this article, or claim that may be made by its manufacturer, is not guaranteed or endorsed by the publisher.
